# Global Blood Pressure Screening During and After Pregnancy: May Measurement Month 2019

**DOI:** 10.1161/HYPERTENSIONAHA.124.23458

**Published:** 2024-09-09

**Authors:** Liza Bowen, Richard J. Stevens, Aletta E. Schutte, Thomas Beaney, Neil R. Poulter, Richard J. McManus, Lucy C. Chappell

**Affiliations:** Population Health Research Institute, St George’s University of London, United Kingdom (L.B.).; Nuffield Department of Primary Care Health Sciences, University of Oxford, United Kingdom (R.J.S., R.J.M.M.).; School of Population Health, University of New South Wales; The George Institute for Global Health, Sydney, Australia (A.E.S.).; Department of Primary Care and Public Health (T.B.), Imperial College London, United Kingdom.; Imperial Clinical Trials Unit, School of Public Health (N.R.P.), Imperial College London, United Kingdom.; Department of Women and Children’s Health, Kings College London, United Kingdom (L.C.C.).

**Keywords:** blood pressure, eclampsia, heart disease risk factors, hypertension, pregnancy

## Abstract

**BACKGROUND:**

Hypertensive disorders of pregnancy are associated with high maternal and fetal morbidity and mortality. There are limited global data on the characteristics of women during and after pregnancy hypertension.

**METHODS:**

May Measurement Month is a global campaign to raise awareness of the importance of blood pressure. Adults (≥18 years) recruited through opportunistic sampling during May 2019 had blood pressure measured and comorbidities and lifestyle data collected. This secondary analysis included 16 519 pregnant women and 529 172 nonpregnant women (16 457 with previous raised blood pressure in pregnancy) from 64 countries.

**RESULTS:**

Almost half of the pregnant women (43.3%) reported not having had their blood pressure measured in the past year, and 14.3% (95% CI, 12.1–16.6) had hypertension (blood pressure ≥140/90 mm Hg or taking antihypertensive medication). Diabetes was self-reported in 7.6% (5.9–9.3) of pregnant women with hypertension and 2.8% (1.9–3.6) of pregnant women without hypertension. In nonpregnant women with and without a history of pregnancy hypertension, age-standardized proportions with current hypertension were 53.2% (50.8–55.7) versus 33.3% (29.3–37.3); with diabetes were 14.4% (11.8–17.0) versus 8.5% (6.3–10.9); and with body mass index ≥30 kg/m^2^ were 28.4% (23.5–33.3) versus 16.6% (13.0–20.2).

**CONCLUSIONS:**

Hypertension in pregnancy was common in this global sample but many cases had not previously been identified. There was a clustering of cardiovascular risk factors in both pregnant women with current hypertension and previously raised blood pressure in pregnancy. This work highlights the importance of screening pregnant women for hypertension, which remains a challenge in large parts of the world.

**NOVELTY AND RELEVANCE**

**What Is New?**

One in 7 of the pregnant women who volunteered for blood pressure measurement in this global sample were found to have hypertension.
Almost half of the pregnant women surveyed had not had their blood pressure measured in the past 12 months.
There was clustering of cardiovascular risk factors in women with hypertension in a current pregnancy compared to pregnant women without hypertension, and in nonpregnant women with a previous history of hypertension in pregnancy, compared to women without such a history.

**What Is Relevant?**

There remains unmet need in screening for hypertension in and after pregnancy, and control of cardiovascular risk factors with an emphasis in low- and middle-income countries.

**Clinical/Pathophysiological Implications?**

Antenatal and postnatal interventions are needed to reduce risks to the woman and fetus in pregnancy, and to reduce long-term cardiovascular disease burden.

Hypertensive disorders of pregnancy (HDP) are associated with high maternal and fetal morbidity and mortality.^1–5^ The most serious complications such as eclampsia and hemolysis, elevated liver enzymes, and low platelets syndrome are more common in low- and middle-income countries compared to high-income countries, thought to be in part due to scarcer availability of antenatal care and later diagnoses.^6–9^

Ongoing initiatives aim to introduce low-cost blood pressure (BP) monitoring equipment and early warning tools to try to improve the early detection of hypertension in pregnancy, but there remains an unmet need and HDP continues to be a leading contributor to maternal mortality worldwide.^10^ Understanding more about the burden of HDP is important in the context of the United Nations Sustainable Development Goal to reduce the global maternal mortality ratio to <70 per 100 000 live births, by 2030.^11^

Modeled data from GBD (Global Burden of Disease) studies provide estimates of maternal mortality from HDP and epidemiological trends in HDP, but there are limited data describing the characteristics of women with a history of HDP globally.^7,12^ Most research work on long-term sequelae of HDP has come from high-income countries.^13–16^ This shows that women with HDP are around twice as likely to subsequently develop hypertension and associated cardiovascular complications.^15,16^

May Measurement Month (MMM) is a global campaign to raise awareness of the importance of BP, initiated by the International Society of Hypertension in 2017 and repeated annually.^17–19^ During the month of May, opportunistic BP screening sites are set up in countries across the world. The data collected during this process offer a unique global cross section of BP readings along with a questionnaire collecting selected demographic and cardiovascular risk factors.

Pregnant women were included in the screening and were the first focus of this secondary analysis, which aimed to describe the characteristics of an opportunistic global sample of pregnant women, along with a comparison of pregnant women with and without hypertension in pregnancy. A further analysis aimed to describe the risk factor profile of screened nonpregnant women with a self-reported history of raised BP in pregnancy compared with those without a self-reported history of raised BP in pregnancy.

## METHODS

### Data Availability

The data used in this study are available for research purposes on approval from the May Measurement Month Management Board. For further details, please visit https://maymeasure.org/about or contact the corresponding author.

### Study Design

MMM is an annual cross-sectional survey of adults (≥18 years) who wished to have their BP measured at any of the MMM screening sites. Full details of the study methodology are available in the main MMM study paper.^17^ These analyses used the 2019 MMM data set (data collected during May 2019) as these data had the largest population of pregnant women and women with a history of raised BP in pregnancy available. For the 2019 MMM, countries were contacted via national societies of hypertension and related conditions or following previous participation in MMM. One or more national leaders were appointed in each country and asked to obtain ethical clearance if required in their jurisdiction. They subsequently set up a network of volunteer investigators who arranged as many local MMM screening sites as possible, in a wide range of locations including health care settings, indoor and outdoor public places, and workplaces. Recruitment methodologies were adopted pragmatically by investigating teams in each region or country.

A common protocol and training materials were provided to volunteer investigators (available on the MMM website: www.maymeasure.org). Those who gave informed consent had their BP measured and completed a short questionnaire (Figure S1). Where facilities were available, weight and height were also measured and recorded. Where facilities were unavailable, weight and height were estimated by those screened. Body mass index was calculated as the weight in kilograms divided by the square of the height in meters. Written instructions and videos on BP measurement were given to all sites. BP was measured 3× while sitting, with measurements taken at 1-minute intervals after participants had been seated for 5 minutes. The majority (85%) of BP readings were taken with OMRON monitors (specific models not recorded).

### Inclusion Criteria

The first analysis included women of reproductive age (18–55 years, upper age limit of 55 based on World Health Organization age of menopause report),^20^ separated into those who did and did not report being currently pregnant. Countries with fewer than 10 pregnant women were excluded.

Within the currently pregnant group, a second analysis compared women with hypertension in pregnancy and without hypertension in pregnancy.

The third analysis included women who were not currently pregnant, comparing those reporting a history of raised BP in pregnancy with those who did not. Countries with fewer than 10 women with a history of raised BP in pregnancy were excluded. To allow for age standardization of results, only participants with data on age were included in analyses. Data cleaning was done centrally according to prespecified criteria.^17^ Geographic regions were defined using the United Nations classification.^21^

### Statistical Analysis

#### Pregnant Population

Descriptive analyses of demographic profile, comorbidities (diabetes, history of myocardial infarction, history of stroke), cardiovascular risk factors, and medication use were looked at in the pregnant population and compared with the nonpregnant population, age standardized to the MMM pregnant population. Analyses were done on the total population and sub-divided by region. The question on diabetes was Do you have diabetes? and did not differentiate between type 1, type 2, and gestational diabetes.

Hypertension was defined as systolic BP≥140 mm Hg or diastolic BP≥90 mm Hg at the time of measurement (using mean of second and third BP readings) or self-reported treatment with antihypertensive medication.^22,23^ Where one or 2 BP readings were missing, multiple imputation using chained equations was conducted centrally by the MMM team as described previously.^17,24,25^ Missingness of BP readings was assumed to be at random, conditional on the remaining variables in the model. Imputed BP measurements were used to calculate mean systolic and diastolic BP and to classify participants into hypertensive/ normotensive status using the thresholds above. Further information on the multiple imputation model is available in the main MMM paper.^17^ The proportion of women with hypertension who self-reported treatment with medication was calculated. Of those on medication for hypertension, the proportion with controlled BP defined as BP <140/90 mm Hg at the MMM screening^26^ was also calculated.

Demographic characteristics of the total population and by region were explored. Analyses of risk factor profiles were stratified by pregnancy status and then by hypertensive status within the pregnant group. Proportions of participants with comorbidities were calculated, along with the proportion reporting alcohol use and tobacco use (noting that the survey was not specific to pregnancy, and the questions asked were simply Do you use tobacco? and Do you consume alcohol? Figure S1).^18^ The proportion who reported having had their BP measured in the past 12 months was also calculated. Analyses were age standardized, using the 2019 MMM pregnant population as the standard reference population.

#### Nonpregnant Women With a History of Raised BP in Pregnancy

A descriptive analysis of comorbidities, risk factors for cardiovascular disease, and medication use was done in the nonpregnant population with a self-reported history of raised BP in pregnancy, compared to the nonpregnant population without a self-reported history of raised BP in pregnancy. Hypertension was defined as described above. Of the women with hypertension, the proportion of women who self-reported a known diagnosis of hypertension (outside of pregnancy, by a health professional) was calculated. The proportion of women with hypertension who self-reported treatment with antihypertensive medication was calculated. Of those on medication for hypertension, the proportion with controlled BP defined as BP <140/90 mm Hg at the MMM screening was also calculated. Mean systolic and diastolic BP were calculated using the imputed data. Analyses were age standardized, using the 2019 MMM population of women as the standard reference population.

Stata version 17 (StataCorp LLC, TX) was used for all analyses.

## RESULTS

### Population

Data on 775 439 women were available from the 2019 MMM database (Figure). Seventeen thousand seven hundred sixty-two women reported being currently pregnant; 1081 of these women were excluded due to reported ages of >55 years,^20^ 91 due to missing data on age, and 71 from countries with fewer than 10 pregnant women. This left data on 16 519 pregnant women for analysis, from 56 countries (Table S1). A comparison group of nonpregnant women from the same countries and in the same age group (18–55) included 465 191 women.

**Figure.**
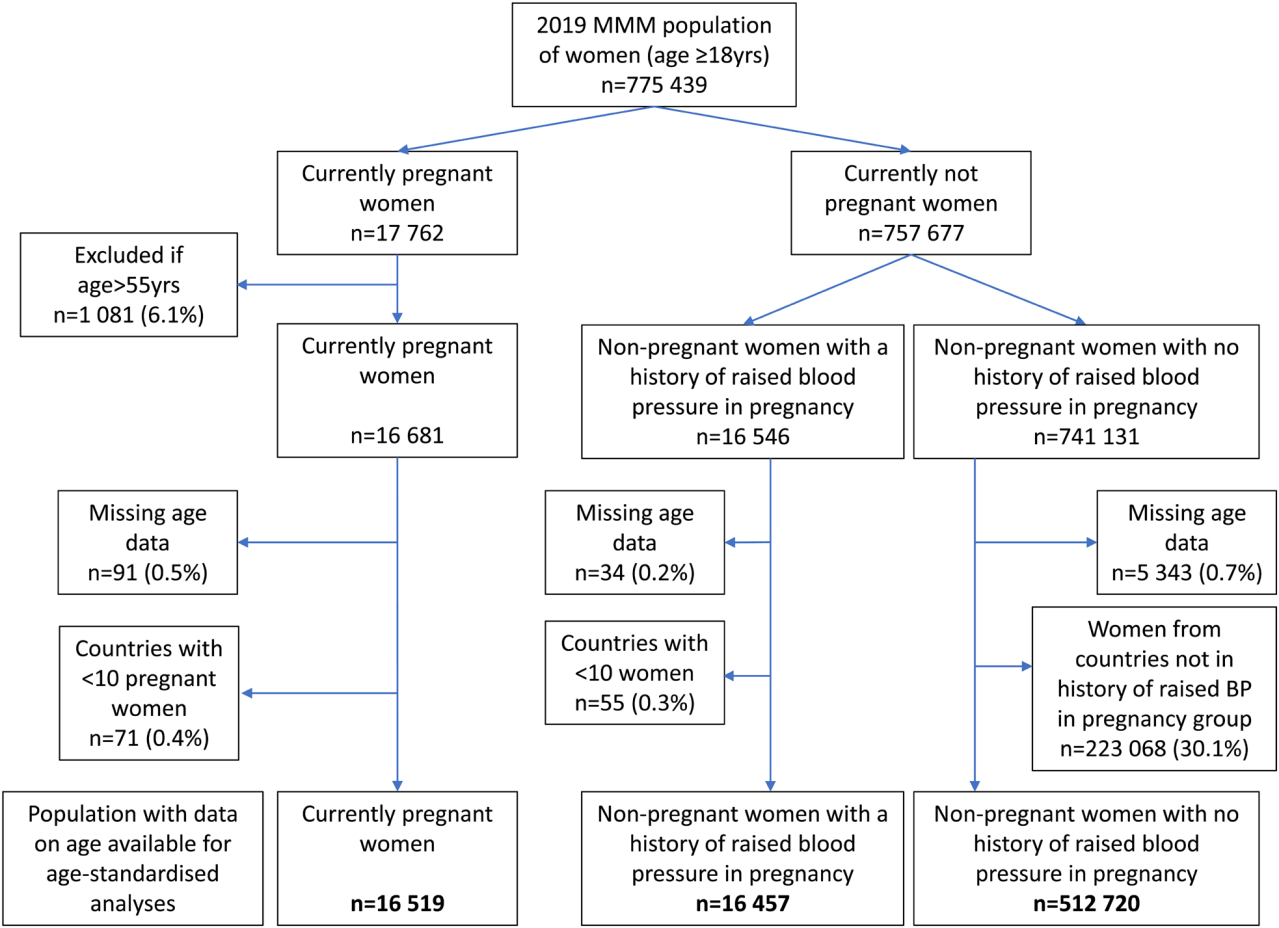
**Flow chart of inclusion in analysis.** MMM indicates May Measurement Month.

Data on 757 677 nonpregnant women were available. Of these, 16 546 (2.3% [95% CI, 1.1–3.6]) reported a history of raised BP in pregnancy, 741 131 did not. Of nonpregnant women, 5377 were excluded due to missing data on age, and 55 women were excluded from countries with fewer than 10 women with a history of raised BP during pregnancy. In the group of women with no history of raised BP in pregnancy, 223 068 women were excluded because they were from countries where there were no women reporting a history of raised BP during pregnancy. This left 16 457 nonpregnant women with a self-reported history of raised BP in pregnancy and 512 720 nonpregnant women with no self-reported history of raised BP in pregnancy for analyses, from 60 countries (Figure; Table S2).

All women included in analyses had at least one BP reading and 515336 (81.2%) had all 3 BP readings recorded.

### Pregnant Population

Participants were screened across a wide geographic spread. The highest proportion was sampled from Asia (60.4%) followed by Africa (25.1%) and the Americas (10.9%; Table 1). The proportion sampled from low- and middle-income countries was 91.8%; 8.2% were sampled from high-income countries.^27^ Globally, 32.8% reported their ethnicity as South Asian, 20.4% as Black, and 10.9% as South-East Asian. Around half (46.7%) of the screening took place in hospitals or clinics, with the remainder being in outdoor public areas (31.3%), indoor public areas (7.1%), workplaces (5.9%), and other/missing (6.6%).

**Table 1.**
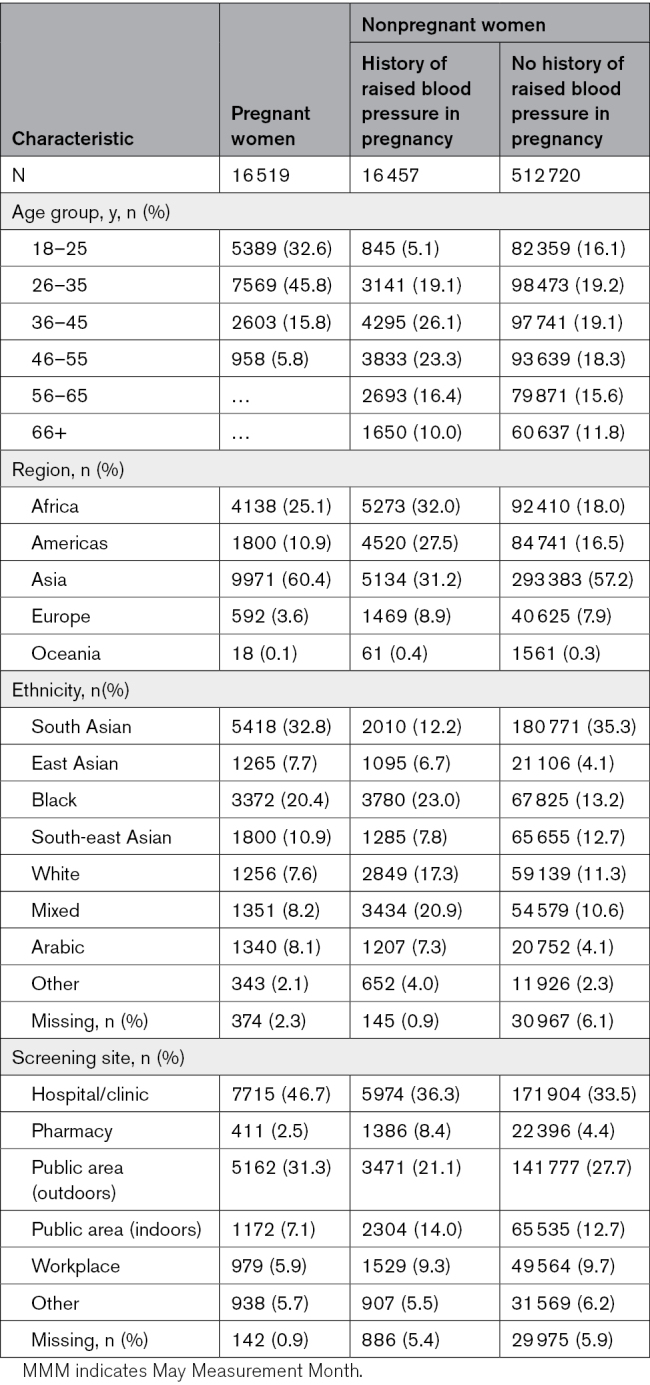
Demographic Characteristics of Pregnant and Nonpregnant Women in the MMM 2019 Population

The prevalence of hypertension (BP≥140/90 mm Hg at time of screening or on antihypertensive medication) in the sample of pregnant women was 14.3% (95% CI, 12.1–16.6; Table 2). Of pregnant women with hypertension, 40.0% (95% CI, 34.5–45.3) reported being on antihypertensive medication, which was similar to the nonpregnant population (39.1% [95% CI, 26.1–52.1]). Of those pregnant women taking medication for hypertension, 67.3% (95% CI, 61.8–72.7) had BP controlled to <140/90 mm Hg at the MMM screening, again similar to the nonpregnant population (65.9% [95% CI, 63.0–68.7]).

**Table 2.**
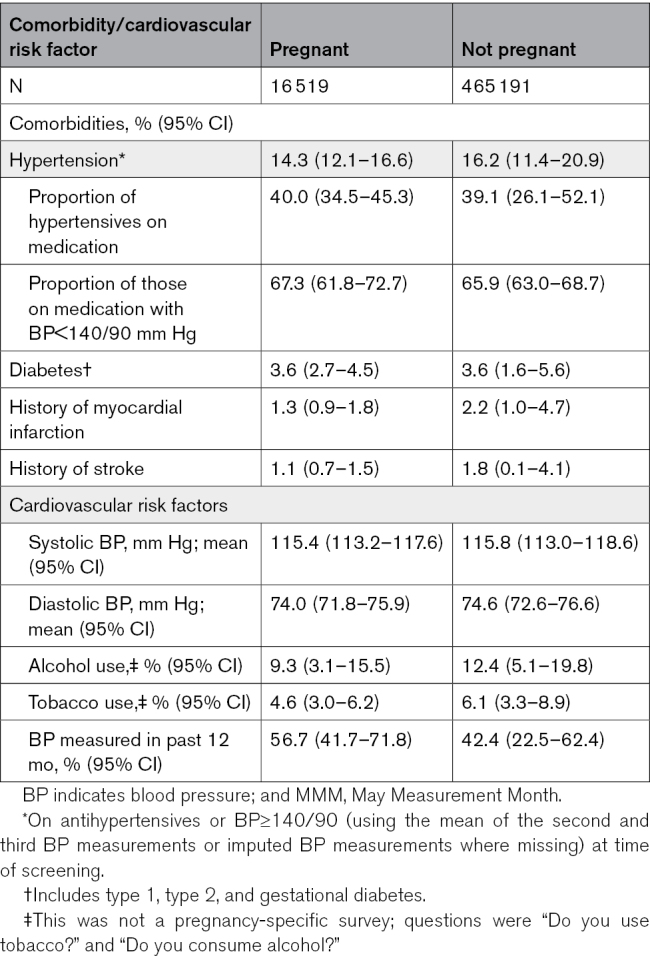
Cardiovascular Risk Profile and Medication Use in Pregnant vs Nonpregnant Women in the MMM 2019 Population (Age Standardized to MMM 2019 Pregnant Population)

Self-reported previous history of diabetes, myocardial infarction, and stroke in the pregnant population was 3.6% (95% CI, 2.7–4.5), 1.3% (95% CI, 0.9–1.8), and 1.1% (95% CI, 0.7–1.5), respectively. Of pregnant women, 9.3% (95% CI, 3.1–15.5) reported some alcohol use, and 4.6% (95% CI, 3.0–6.2) reported tobacco use (Table 2).

Globally 56.7% (95% CI, 41.7–71.8) of pregnant women reported having their BP measured in the past year, ranging from 51.9% (95% CI, 31.1–72.1) in Asia to 78.1% (95% CI, 71.5–83.5) in Europe (Table S3).

The reported prevalence of all comorbidities was higher in the population of pregnant women with hypertension than those without pregnancy hypertension, for example, 7.6% (95% CI, 5.9–9.3) of women with hypertension in pregnancy reported having diabetes compared with 2.8% (95% CI, 1.9–3.6) in pregnant women without hypertension (Table 3). This pattern was consistent across regions (Table S4).

**Table 3.**
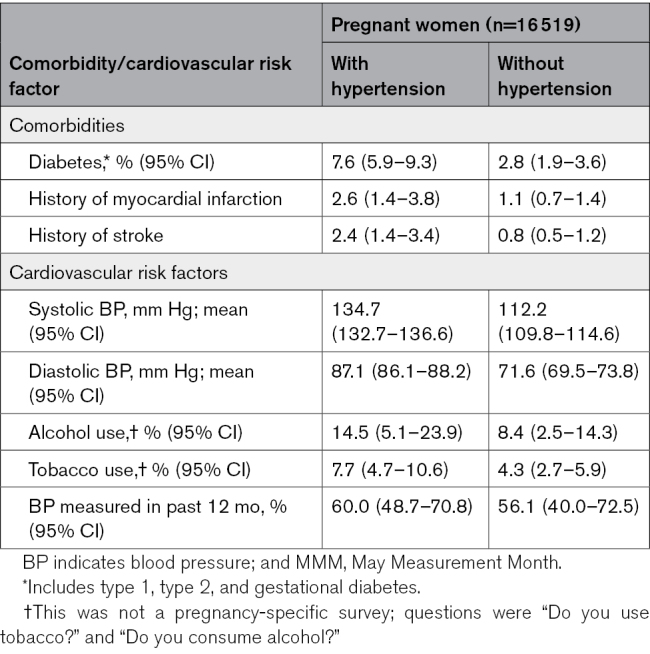
Cardiovascular Risk Profile in the Pregnant MMM 2019 Population in Those Who Have Hypertension (See Text for Details) and in Those Who Have No Hypertension (Age Standardized to MMM 2019 Total Pregnant Population)

### Nonpregnant Women (With and Without a History of Raised BP in Pregnancy)

Of nonpregnant women in the population, 89.6% were from low- and middle-income countries and 10.4% were from high income countries.^27^ Globally, over half of the women reporting a background of raised BP in pregnancy had hypertension at the time of study (53.2% [95% CI, 50.8–55.7]), of whom 80.0% (76.7–83.3) were aware of a diagnosis of hypertension (outside of pregnancy) and 69.5% (64.9–74.1) were on antihypertensive medication (Table 4). In nonpregnant women without a history of raised BP in pregnancy, 33.3% had hypertension at the time of the study (95% CI, 29.3–37.3), of whom 58.0% (48.9–67.0) were aware of a diagnosis of hypertension (outside pregnancy) and 61.1% (57.7–64.5) were on antihypertensive medication.

**Table 4.**
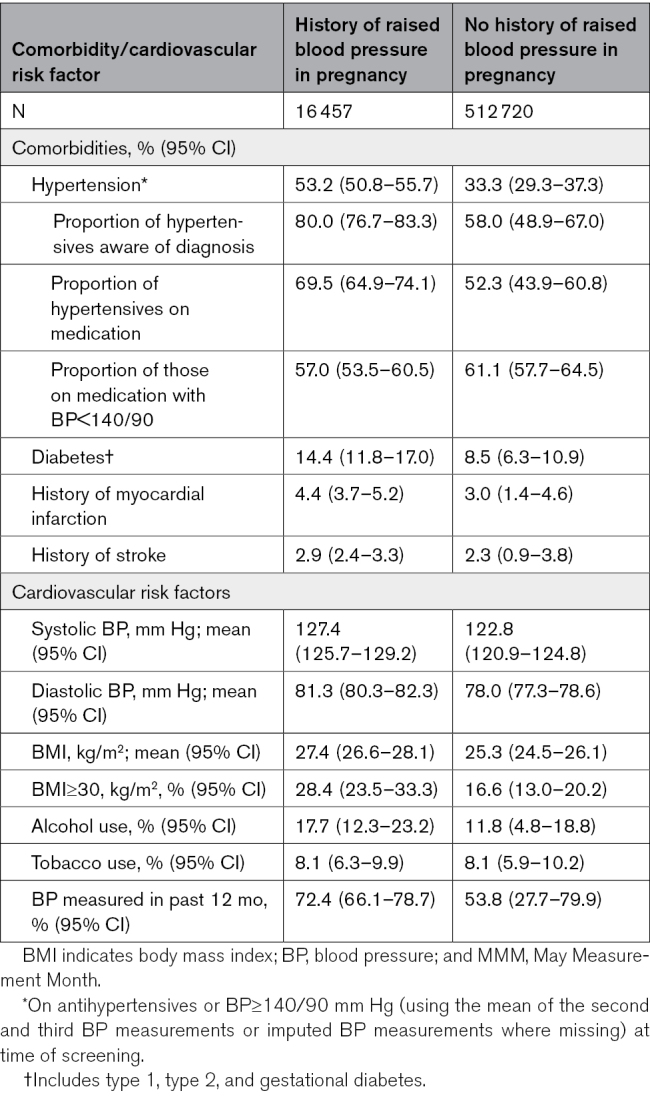
Cardiovascular Risk Factor Profile and Medication Use in Women in the MMM 2019 Nonpregnant Population Reporting a History of Raised Blood Pressure in Pregnancy Versus Those Not Reporting a History of Raised Blood Pressure in Pregnancy (Age Standardized to MMM 2019 Population of Women)

Diabetes was reported in 14.4% (95% CI, 11.8–17.0) of women with a history of raised BP in pregnancy and in 8.5% (6.3–10.9) of women without a history of raised BP in pregnancy. Body mass index ≥30 kg/m^2^ was seen in 28.4% (95% CI, 23.5–33.3) women with a history of raised BP in pregnancy and in 16.6% (13.0–20.2) of women without a history of raised BP in pregnancy. This pattern was consistent across regions (Table S5).

## DISCUSSION

### Main Findings

In this global sample of 16 519 pregnant women from 56 countries, 14.3% were found to have hypertension. Fewer than half (40%) of these pregnant women with hypertension were on medication. This is likely to be due to a combination of women not having had their BP monitored in this pregnancy (almost half of the pregnant women reported not having had their BP checked in the past year), women who had previously been monitored in this pregnancy but were found to have high BP for the first time at the MMM screening, and women who have had previously high readings but not been put on medication. These data highlight high rates of inadequate detection and management of hypertension in pregnancy. A large percentage of the pregnant women in this study (>90%) were from low- and middle-income countries HDP continue to be one of the major causes of maternal mortality worldwide and the lack of BP checks and potential for early treatment may be contributing to this.^28^

There was a clustering of comorbidities, with higher reported prevalence of known diabetes and history of myocardial infarction and stroke in the pregnant women with hypertension than without, which has been seen in other studies.^29–32^

In nonpregnant women with a history of raised BP in pregnancy, there was a higher burden of cardiovascular risk factors and higher mean BP than in nonpregnant women without such a history. Over half of women with a history of raised BP in pregnancy were hypertensive, nearly a third had a body mass index ≥30 kg/m^2^, and 14.4% reported having diabetes.

### Previous Literature

Large, global studies of hypertension in pregnancy are uncommon. A recent analysis of GBD data used modeled data from the Global Health Data Exchange database and found a global age-standardized incidence rate of HDP of 463 per 100 000 population in 2019.^12^ Incidence rates of HDP were the highest in Western sub-Saharan Africa (1615 per 100 000 population), Central sub-Saharan Africa (1518 per 100 000 population) and Eastern sub-Saharan Africa (1496 per 100 000 population), and the lowest in East Asia (98 per 100 000 population), high income Asia pacific (144 per 100 000 population), and Central Europe (161 per 100 000 population). The GBD data used a combination of diverse sources of data including national registries and local surveys, and estimates are likely to be more representative where there are more extensive data collection and less representative in low- and middle-income countries where there will be greater reliance on smaller studies. It is difficult to make direct comparisons between results from the GBD study and the MMM data presented here due to the different measures used. There was also limited information on broader risk factor profiles of the women with HDP included in the GBD analyses.

Many estimates of HDP prevalence are around 10%, but the largest data sources have been from high-income countries, and estimates in low-income settings are often from small hospital surveys that may not be representative.^3,4,33–35^ A recent prospective population-based study in India, Pakistan, and Mozambique estimated higher prevalence of 14.0%, 11.6%, and 16.8%, respectively when a combination of study BP measurements and household surveys/facility record reviews of diagnoses was used.^36^ These latter estimates are closer to the 14.3% found in the MMM study population, where the majority (85%) of the pregnant women were from Africa and Asia.

### Strengths and Limitations

A significant strength of this study was the large sample size and geographically diverse population of both pregnant women and nonpregnant women with a history of raised BP in pregnancy, with a large proportion (≈90%) from low- and middle-income countries. It also includes women sampled from settings outside clinics and hospitals that may otherwise be rarely represented.

A limitation is the convenience sampling method, meaning that the women screened are unlikely to be representative of the population of women in the countries included. There was a high proportion of pregnant women from Asia and Africa, and within each region, there were some subregions with greater numbers than others. This should be taken into account when considering the regional estimates and has implications for making cross-regional comparisons or considering estimates as representative of regional prevalence. It is also possible that there have been some changes in access to BP measurement and disease burden since the data were collected in 2019; while it is possible that access to measurement has increased, it is most likely that access reduced following COVID.

The proportion classified as hypertensive should be interpreted with caution as there may be some selection bias in women presenting to be screened. Women who are high risk or have known hypertension may be more motivated to have their BP checked, and the hospital/clinic screening sites will be more frequently encountered by women with hypertension than women with uncomplicated pregnancies who have fewer appointments. Although the majority of BP monitors used were OMRON devices (many of which are pregnancy-validated), it was not possible to confirm which models were used and therefore whether they were specifically validated for use in pregnancy.^37^ Last, the BP readings used to define diagnosis were from a single time point only. However, even transient high BP is important as it is associated with an increased risk of developing sustained gestational hypertension or preeclampsia in the remainder of the pregnancy,^38^ or may indicate undiagnosed chronic hypertension.

Pregnancy status was based on self-report and is therefore subject to potential bias. We excluded any pregnancies recorded in women with an age of >55 years (6.1% of pregnancies) as this was thought unlikely to be a true pregnancy but we cannot exclude some level of misclassification in pregnancy status in women aged <55 years. A sensitivity analysis restricted to pregnant women ≤45 years did not make any meaningful difference to the results (Tables S6 and S7).

When interpreting the data on the proportion of pregnant women reporting having had their BP measured in the past 12 months, it is important to acknowledge that we do not know the gestation of pregnancy and cannot distinguish women with early stage pregnancies who we would not expect to have had an antenatal care booking visit yet, from women with later stage pregnancies.

The classification of women with a history of raised BP in pregnancy is also subject to potential measurement error as it was based on self-report. There may also be a survival effect due to the cross-sectional design, with women with raised BP in pregnancy at greater risk of mortality during and after the perinatal period. However, we would expect these numbers to be small and to have reduced the estimates of comorbidities and cardiovascular risk factors in the group classified as having a history of raised BP in pregnancy; therefore, underestimating any differences seen when comparing to the group classified as having no history of raised BP in pregnancy.

### Perspectives

These data provide a quantification and description of global populations of both pregnant women, and nonpregnant women with a history of raised BP in pregnancy. There remains unmet need in both under-measurement of BP in pregnancy, regular measurement of BP in women who have a history of raised BP in pregnancy, and control of risk factors associated with greater morbidity and mortality in a population with current or previous raised BP in pregnancy. Future work should look at how perinatal and postnatal interventions could consider the clustering of comorbidities and improve the risk factor profile of women across the life course to ameliorate the longer-term cardiovascular disease burden.

## ARTICLE INFORMATION

### Acknowledgments

The authors thank all May Measurement Month (MMM) volunteer staff and participants.

### Author Contributions

T. Beaney, N. Poulter, and A.E. Schutte were involved in the conception and running of the MMM (May Measurement Month) study. The protocol for this analysis was developed by all authors. The analysis was completed by L. Bowen. The first draft of the paper was written by L. Bowen with input from all authors.

### Sources of Funding

May Measurement Month (MMM) is an initiative of the International Society of Hypertension. Servier supported the study through Institut la Conference Hippocrate but had no role in study design, data collection, data analysis, data interpretation, or writing of the report. R.J. McManus and L.C. Chappell have been supported by Research Professorships from the National Institute of Health Research (NIHR-RP-R2-12-015 and RP-2014-05-019 respectively) and are now National Institute of Health Research Senior Investigators. R.J. McManus holds a current National Institute for Health Research Programme Grant for Applied Research (RP-PG-0614-20005). R.J. McManus has received funding from the National Institute for Health Research Applied Research Collaboration Oxford and Thames Valley. A.E. Schutte is supported by a National Health and Medical Research Council Investigator Grant (APP2017504). L. Bowen is supported by a National Institute of Health Research Clinical Lectureship in General Practice (CL-2022-16-001). The views expressed in this publication are those of the authors and not necessarily those of the National Health Service, the National Institute of Health Research or the Department of Health. N. Poulter was supported by the National Institute for Health Research Senior Investigator Awards, Biomedical Research Centre funding, and the British Heart Foundation Research Centre Excellence Award. The first author had full access to all the data in the study and had final responsibility for the decision to submit for publication.

### Disclosures

A.E. Schutte has received speaker honoraria, consultancy fees or both from Omron, Aktiia, Abbott, Servier, Medtronic, Sanofi, Novartis, and Skylabs. N. Poulter has received financial support from several pharmaceutical companies, which manufacture BP-lowering agents, for consultancy fees (Servier, Aktiia), research projects and staff (Servier, Pfizer) and for arranging and speaking at educational meetings (AstraZeneca, Lri Therapharma, Napi, Servier, Sanofi, Eva Pharma, Pfizer, Emcure India, Dr Reddy’s Laboratories). He holds no stocks and shares in any such companies. R.J. McManus has worked with Omron and Sensyne to develop telemonitoring solutions for which his institution has received licencing and consultancy payments.

### Supplemental Material

Title page

Text

Reference Section

Tables S1–S7

Figure S1

## Supplementary Material


