# Supplementary material for: Global Blood Pressure Screening During and After Pregnancy: May Measurement Month 2019
Source: Hypertension. 2024 Sep 9;81(11):2298–306. doi: 10.1161/HYPERTENSIONAHA.124.23458 (PMC11485200; doi:10.1161/HYPERTENSIONAHA.124.23458)

**Supplemental Material**

Title: Global blood pressure screening during and after pregnancy: May Measurement Month 2019

Liza Bowen^a^, Richard Stevens^b^, Aletta E Schutte^c^, Thomas Beaney^d^, Neil Poulter^e^, Richard J McManus^b^, Lucy C Chappell^f^

^a^ Population Health Research Institute, St George’s University of London, UK

^b^ Nuffield Department of Primary Care Health Sciences, University of Oxford, UK

^c^ School of Population Health, University of New South Wales; The George Institute for Global Health, Sydney, Australia

^d^ Department of Primary Care and Public Health, Imperial College London, UK

^e^ Imperial Clinical Trials Unit, School of Public Health, Imperial College London, UK

^f^ Department of Women and Children’s Health, Kings College London, UK

Short Title: Global pregnancy hypertension screening

**Corresponding Author:** Dr Liza Bowen; [lbowen@sgul.ac.uk](mailto:lbowen@sgul.ac.uk); Population Health Research Institute, St George’s University of London, Cranmer Terrace, London SW17 0RE, [020 8672 9944](https://www.google.com/search?q=st+george%27s+university+of+london&rlz=1C1CHBD_en-GBGB1040GB1040&oq=st+george%27s+&aqs=chrome.1.69i57j69i59l2j0i271l3.3927j0j1&sourceid=chrome&ie=UTF-8)

**Contents**

Supplementary text

Supplemental reference section

Tables S1–S7
Figure S1

**Supplementary text**

**Multiple imputation**

Multiple imputation was done centrally by the MMM team, as described previously^1^. Data on a total of 1,508,130 participants were collected during MMM19, with 75.1% having three BP readings. To provide comparable readings across all individuals, including those where fewer than three readings were recorded, multiple imputation by chained equations (MICE) was used, which assumed missingness was Missing-at-Random (MAR) and therefore dependent on the observed data only (and not on the unobserved values). Multiple imputation was conducted using the mi impute chained routing in Stata for those with complete data on age, gender (male or female, and not equal to other), ethnicity and anti-hypertensive medication status. The complete imputation model included the following variables: gender, age (as a restricted cubic spline with 5 knots), gender by age interaction, ethnicity, anti-hypertensive medication status, known hypertension, previous participation in MMM (2017/2018), last blood pressure measurement, region, country income status, screening site type, height, weight, diabetes, stroke, myocardial infarction, smoking status, aspirin use, statin use, current pregnancy status, previous hypertension in pregnancy, fasting status, day of week, alcohol consumption, along with each of the three systolic BP, diastolic BP and heart rate measurements, and the corresponding mean of the second and third readings.

Therefore, following guidance provided by White et al the imputation model included all variables selected to be in the main MMM analysis.^2^ Variables which were used to compute the variables within the analysis models (e.g. sbp1, sbp2, sb3, dbp1, dbp2, dbp3, hr1, hr2, hr3) were also included following the just another variable (JAV) approach as described by Seaman et al.^3^

A total of 25 imputations were created, corresponding to just over one imputation per percent missing data on the mean of the second and third blood pressure measures (20% missingness). Following imputation we assessed the Monte Carlo error of estimates to confirm this gave adequate precision. The Monte Carlo errors of estimates were <10% of their standard errors indicating sufficient precision. A burn-in of 10 iterations was chosen for each imputation chain meaning that imputed data sets were stored at every 10th iteration of the chain. An assessment of the predicted values from each iteration in a trace pot confirmed this was adequate and chains had converged. Results of analyses on the imputed data were pooled across imputed data sets using Rubin’s combination rules.

For individuals missing second and/or third blood pressure reading for whom the mean of second and third systolic blood pressure measurement could not be imputed using the full MI model (due to also missing one or more of age, gender, ethnicity and anti-hypertensive medication status or gender of ‘other’ recorded) we used a reduced MI model, which included each systolic and diastolic BP reading, along with the mean of the second and third readings. Thus, following imputation, an estimate of the mean of the second and third BP reading was available for all participants.

**Supplementary References**

1. Beaney T, Schutte AE, Stergiou GS, Borghi C, Burger D, Charchar F, Cro S, Diaz A, Damasceno A, Espeche W, et al. May Measurement Month 2019. *Hypertension*. 2020;76:333-341. doi: doi:10.1161/HYPERTENSIONAHA.120.14874

2. White IR, Royston P, Wood AM. Multiple imputation using chained equations: Issues and guidance for practice. *Stat Med*. 2011;30:377-399. doi: 10.1002/sim.4067

3. Seaman SR, Bartlett JW, White IR. Multiple imputation of missing covariates with non-linear effects and interactions: an evaluation of statistical methods. *BMC Med Res Methodol*. 2012;12:46. doi: 10.1186/1471-2288-12-46

**Supplemental tables**

**Table S1: Countries from which pregnant women and non-pregnant comparison group (age 18-55) were screened**

| Country | Pregnant | Non-pregnant |
| --- | --- | --- |
|  |  |  |
| Albania | 257 | 9,824 |
| Angola | 111 | 2,855 |
| Armenia | 279 | 3,442 |
| Australia | 18 | 1,062 |
| Bangladesh | 688 | 10,242 |
| Benin | 58 | 1,613 |
| Bosnia and Herzegovina | 42 | 599 |
| Botswana | 44 | 2,719 |
| Brazil | 128 | 5,333 |
| Bulgaria | 10 | 415 |
| Cabo Verde | 695 | 8,135 |
| Cameroon | 747 | 12,338 |
| Chile | 77 | 2,837 |
| China | 1,079 | 80,857 |
| Colombia | 637 | 18,431 |
| Democratic Republic of Congo | 863 | 8,871 |
| Dominican Republic | 31 | 700 |
| Ecuador | 55 | 7,695 |
| Georgia | 33 | 3,786 |
| Ghana | 84 | 2,959 |
| Guatemala | 52 | 742 |
| Honduras | 30 | 930 |
| Hungary | 20 | 1,065 |
| India | 3,738 | 112,732 |
| Jamaica | 23 | 1,086 |
| Kenya | 532 | 15,248 |
| Kyrgyzstan | 18 | 861 |
| Lebanon | 113 | 1,972 |
| Libya | 298 | 3,291 |
| Malawi | 94 | 3,059 |
| Malaysia | 31 | 1,679 |
| Mauritius | 42 | 3,162 |
| Mexico | 442 | 14,495 |
| Mongolia | 87 | 1,834 |
| Nepal | 969 | 25,145 |
| Nigeria | 44 | 1,412 |
| Oman | 11 | 387 |
| Pakistan | 179 | 1,624 |
| Paraguay | 65 | 1,820 |
| Philippines | 1,487 | 33,429 |
| Poland | 56 | 2,046 |
| Portugal | 30 | 634 |
| Republic of Korea | 19 | 2,184 |
| Russia | 57 | 2,706 |
| South Africa | 57 | 2,251 |
| Spain | 45 | 1,276 |
| Sri Lanka | 52 | 709 |
| Switzerland | 18 | 624 |
| Tunisia | 277 | 4,384 |
| United Arab Emirates | 1,038 | 13,504 |
| United Kingdom | 24 | 3,057 |
| Uruguay | 10 | 445 |
| Venezuela | 250 | 7,478 |
| Vietnam | 183 | 8,926 |
| Zambia | 167 | 3,973 |
| Zimbabwe | 25 | 308 |
|  |  |  |
| Total | 16,519 | 465,191 |

**Table S2: Countries from which non-pregnant women with a history of raised blood pressure in pregnancy and comparison group of non-pregnant women without a history of raised blood pressure in pregnancy were screened**

| Country | History of raised BP in pregnancy | No history of raised BP in pregnancy |
| --- | --- | --- |
|  |  |  |
| Albania | 470 | 12,242 |
| Angola | 444 | 3,023 |
| Armenia | 319 | 5,392 |
| Australia | 61 | 1,561 |
| Bangladesh | 246 | 11,479 |
| Barbados | 13 | 166 |
| Belgium | 80 | 860 |
| Benin | 221 | 1,953 |
| Bosnia and Herzegovina | 69 | 807 |
| Botswana | 280 | 2,770 |
| Bulgaria | 44 | 823 |
| Cabo Verde | 1,203 | 9,329 |
| Cameroon | 611 | 13,842 |
| Canada | 26 | 462 |
| Chile | 283 | 3,841 |
| Colombia | 832 | 24,979 |
| Democratic Republic of Congo | 276 | 10,765 |
| Dominican Republic | 100 | 830 |
| Ecuador | 339 | 12,825 |
| France | 11 | 500 |
| Georgia | 42 | 8,083 |
| Ghana | 86 | 3,486 |
| Guatemala | 83 | 938 |
| Honduras | 38 | 1,163 |
| Hungary | 86 | 1,575 |
| India | 1,067 | 147,407 |
| Jamaica | 267 | 1,492 |
| Kenya | 326 | 19,056 |
| Kyrgyzstan | 38 | 947 |
| Lebanon | 111 | 2,639 |
| Libya | 738 | 3,611 |
| Lithuania | 44 | 258 |
| Malawi | 63 | 3,930 |
| Malaysia | 68 | 1,847 |
| Mauritius | 46 | 4,714 |
| Mexico | 741 | 21,268 |
| Mongolia | 245 | 1,751 |
| Mozambique | 64 | 502 |
| Nepal | 804 | 29,031 |
| Nigeria | 107 | 1,767 |
| Oman | 12 | 452 |
| Paraguay | 233 | 2,330 |
| Philippines | 1,145 | 44,975 |
| Poland | 54 | 4,297 |
| Portugal | 66 | 1,220 |
| Republic of Korea | 42 | 5,674 |
| Russia | 312 | 3,156 |
| South Africa | 170 | 2,834 |
| Sri Lanka | 73 | 906 |
| Switzerland | 31 | 890 |
| Taiwan | 604 | 11,518 |
| Tunisia | 339 | 6,180 |
| Ukraine | 13 | 413 |
| United Arab Emirates | 269 | 14,796 |
| United Kingdom | 147 | 5,501 |
| Uruguay | 61 | 688 |
| Venezuela | 1,504 | 13,759 |
| Vietnam | 91 | 14,569 |
| Zambia | 247 | 4,337 |
| Zimbabwe | 52 | 311 |
|  |  |  |
| Total | 16,457 | 512,720 |

**Table S3: Cardiovascular risk profile and medication use in pregnant vs non-pregnant women in the MMM 2019 population (age standardised to MMM 2019 pregnant population), by region**

| **Comorbidity/ cardiovascular risk factor** | **Africa** | | **Americas** | | **Asia** | | **Europe** | |
| --- | --- | --- | --- | --- | --- | --- | --- | --- |
|  | **Pregnant** | **Not pregnant** | **Pregnant** | **Not pregnant** | **Pregnant** | **Not pregnant** | **Pregnant** | **Not pregnant** |
| **N** | 4 138 | 76 578 | 1 800 | 61 992 | 9 971 | 299 527 | 592 | 26 032 |
| **Comorbidities,** % (95% CI) |  |  |  |  |  |  |  |  |
| Hypertension* | 15.4  (12.9, 17.9) | 15.5  (13.6, 17.4) | 17.6  (14.1, 21.1) | 15.7  (9.2, 22.3) | 13.3  (9.5, 17.2) | 16.6  (9.6, 23.9) | 16.1  (10.6, 21.6) | 12.1  (9.9, 14.2) |
| Proportion of hypertensives on medication | 35.8  (24.1, 47.4) | 25.1  (18.1, 32.0) | 44.2  (31.0, 57.3) | 39.7  (23.7, 55.8) | 40.0  (33.2, 46.8) | 42.8  (24.7, 60.8) | 51.7  (32.7, 70.7) | 29.8  (16.5, 43.2) |
| Proportion of those on medication with BP<140/90 | 57.8  (45.0, 70.6) | 63.8  (59.1, 68.6) | 77.2  (71.1, 83.3) | 74.5  (71.9, 77.1) | 68.0  (60.9, 75.1) | 64.5  (62.6, 66.5) | 79.7  (70.5, 88.9) | 75.0  (70.0, 80.0) |
| Diabetes† | 3.5  (2.1, 5.5) | 1.9  (1.3, 2.6) | 4.1  (3.0, 5.4) | 3.5  (1.6, 7.7) | 3.6  (2.6, 5.1) | 4.2  (2.3, 7.9) | 3.8  (2.5, 5.2) | 1.4  (0.2, 1.9) |
| History of myocardial infarction | 1.6 (0.9, 2.8) | 1.0 (0.7, 1.4) | 1.2 (0.8, 1.7) | 1.3 (0.6, 2.6) | 1.3 (0.8, 2.0) | 2.8 (1.3, 6.1) | 0.2 (0.0, 0.8) | 0.3 (0.1, 0.5) |
| History of stroke | 0.9 (0.5, 1.9) | 1.1 (0.4, 2.7) | 2.3 (0.7, 7.3) | 0.6 (0.5, 0.8) | 1.0 (0.6, 1.6) | 2.3 (1.0, 5.3) | 0.6 (0.1, 2.4) | 0.3 (0.1, 0.6) |
| **Cardiovascular risk factors** |  |  |  |  |  |  |  |  |
| Systolic BP (mm Hg), mean (95% CI) | 115.9  (114.3, 117.5) | 116.9  (115.6, 118.1) | 113.5  (110.9, 116.3) | 114.6  (112.1, 117.1) | 115.6  (112.0, 119.2) | 115.8  (111.5, 120.1) | 115.4  (113.3, 117.5) | 114.9  (113.9, 116.0) |
| Diastolic BP (mm Hg), mean (95% CI) | 73.5  (72.0, 75.0) | 75.7  (74.6, 76.6) | 73.6  (71.8, 75.5) | 74.6  (73.6, 75.5) | 73.9  (70.6, 77.3) | 74.3  (71.2, 77.5) | 73.4  (71.7, 75.2) | 74.6  (73.9, 75.3) |
| Alcohol use‡, % (95%CI) | 21.5  (12.2, 35.0) | 27.4  (16.8, 41.3) | 11.1  (6.6, 17.9) | 24.1  (17.9, 31.6) | 3.5 (1.3, 9.2) | 5.1 (2.4, 10.1) | 11.5  (6.1, 20.9) | 28.9  (19.4, 40.6) |
| Tobacco use‡, % (95%CI) | 3.0 (1.9, 4.6) | 3.1 (2.2, 4.1) | 5.2 (4.0, 6.7) | 9.3 (5.5, 15.1) | 5.0 (3.0, 8.3) | 5.8 (2.9, 11.4) | 6.5 (3.3, 12.1) | 13.0  (8.7, 18.9) |
| **BP measured in past 12 months,** % (95%CI) | 57.6  (40.1, 73.3) | 49.4  (39.7, 59.2) | 75.3  (68.9, 80.8) | 66.0  (61.5, 70.2) | 51.9  (31.1, 72.1) | 36.9  (16.1, 62.2) | 78.1  (71.5, 83.5) | 62.8  (51.2, 73.0) |

*on antihypertensives or BP≥140/90 (using the mean of the second and third BP measurements or imputed BP measurements where missing) at time of screening

†includes type1, type 2, and gestational diabetes

‡This was not a pregnancy specific survey; questions were “Do you use tobacco?” and “Do you consume alcohol?”

**Table S4: Cardiovascular risk profile in the pregnant MMM 2019 population in those who have hypertension (see text for details) and in those who have no hypertension (age standardised to MMM 2019 total pregnant population), by region**

| **Comorbidity/ cardiovascular risk factor** | **Africa**  (n=4,138) | | **Americas**  (n=1,800) | | **Asia**  (n=9,971) | | **Europe**  (n=592) | |
| --- | --- | --- | --- | --- | --- | --- | --- | --- |
|  | HTN | No HTN | HTN | No HTN | HTN | No HTN | HTN | No HTN |
| **Comorbidities** |  |  |  |  |  |  |  |  |
| Diabetes*, % (95% CI) | 7.8 (4.0, 11.2) | 2.7 (1.4, 4.1) | 8.1 (5.2, 11.1) | 2.8 (1.5, 4.0) | 7.8 (5.2, 10.3) | 2.8 (1.6, 4.0) | 4.5 (0.4, 8.5) | 3.5 (2.0, 5.0) |
| History of myocardial infarction | 3.6 (0.5, 6.8) | 1.3 (0.7, 1.9) | 2.6 (1.0, 4.2) | 0.7 (0.4, 1.1) | 2.4 (1.2, 3.6) | 1.1 (0.6, 1.6) | No obs‡ | No obs‡ |
| History of stroke | 3.1 (1.1, 5.1) | 0.4 (0.2, 0.7) | No obs‡ | No obs‡ | 2.5 (1.1, 3.8) | 0.8 (0.4, 1.1) | No obs‡ | No obs‡ |
| **Cardiovascular risk factors** |  |  |  |  |  |  |  |  |
| Systolic BP (mm Hg), mean (95% CI) | 138.8  (135.2, 142.4) | 111.8  (110.1, 113.5) | 132.3  (128.7, 136.0) | 109.5  (106.9, 112.2) | 113.5  (131.7, 135.2) | 112.8  (109.3, 116.4) | 131.3  (127.0, 135.7) | 112.3  (110.8, 113.9) |
| Diastolic BP (mm Hg), mean (95% CI) | 88.2  (86.3, 90.2) | 71.0  (69.7, 72.3) | 87.2  (83.2, 91.2) | 70.8  (69.0, 72.7) | 86.9  (85.6, 88.2) | 72.0  (68.7, 75.3) | 83.1  (79.6, 86.6) | 71.5  (69.9, 73.1) |
| Alcohol use†**,** % (95% CI) | 26.3  (16.0, 36.8) | 20.7  (9.0, 32.5) | 17.1  (8.4, 25.8) | 9.9  (4.6, 15.2) | 7.4  (0.0, 16.0) | 2.8  (0.2, 5.5) | 16.4  (5.6, 26.7) | 10.2  (2.7, 17.7) |
| Tobacco use†**,** % (95% CI) | 6.8  (3.0, 10.5) | 2.6  (1.5, 3.7) | 8.5  (4.4, 12.7) | 4.4  (3.4, 5.5) | 7.8  (2.8, 12.8) | 4.7  (2.2, 7.2) | 7.5  (3.0, 12.0) | 6.1  (2.1, 10.1) |
| **BP measured in past 12 months,** % (95% CI) | 47.5  (30.1, 64.0) | 59.1  (42.0, 76.2) | 70.3  (62.8, 77.8) | 76.0  (70.3, 81.6) | 62.6  (46.7, 78.5) | 50.4  (27.4, 73.3) | 78.7  (69.0, 88.4) | 78.5  (72.5, 84.4) |

*includes type1, type 2, and gestational diabetes

†This was not a pregnancy specific survey; questions were “Do you use tobacco?” and “Do you consume alcohol?”

‡No/too few cases to generate estimations

**Table S5: Cardiovascular risk factor profile and medication use in the MMM 2019 non-pregnant population reporting a history of raised blood pressure in pregnancy vs those not reporting a history of raised blood pressure in pregnancy (age standardised to MMM 2019 population of women), by region**

| **Comorbidity/ cardiovascular risk factor** | **Africa** | | **Americas** | | **Asia** | | **Europe** | |
| --- | --- | --- | --- | --- | --- | --- | --- | --- |
|  | **History of raised BP in pregnancy** | **No history of raised BP in pregnancy** | **History of raised BP in pregnancy** | **No history of raised BP in pregnancy** | **History of raised BP in pregnancy** | **No history of raised BP in pregnancy** | **History of raised BP in pregnancy** | **No history of raised BP in pregnancy** |
| **N** | 5 273 | 92 410 | 4 520 | 84 741 | 5 134 | 293 383 | 1 469 | 40 625 |
| **Comorbidities,** % (95% CI) |  |  |  |  |  |  |  |  |
| Hypertension* | 55.3  (50.7, 59.8) | 32.7  (29.9, 35.5) | 50.6  (45.8, 55.3) | 30.4  (24.6, 36.2) | 52.3  (48.1, 56.5) | 33.7  (27.3, 40.1) | 55.3  (51.3, 59.4) | 36.0  (28.9, 43.1) |
| Proportion of hypertensives aware of diagnosis | 77.4  (71.1, 83.6) | 50.0  (42.7, 57.4) | 85.4  (82.2, 88.6) | 63.7  (56.7, 70.7) | 78.9  (72.9, 84.9) | 57.3  (43.6, 71.1) | 75.9  (68.0, 83.7) | 61.9  (49.4, 74.3) |
| Proportion of hypertensives on medication | 61.8  (55.9, 67.6) | 38.6  (32.3, 45.0) | 77.0  (72.3, 81.7) | 55.8  (45.2, 66.3) | 71.2  (61.5, 80.9) | 54.1  (40.8, 67.3) | 67.5  (60.8, 74.1) | 54.9  (40.2, 69.5) |
| Proportion of those on medication with BP<140/90 | 48.6  (43.3, 53.8) | 55.3  (50.5, 60.0) | 64.0  (59.3, 68.7) | 69.9  (66.8, 73.0) | 60.0  (55.1, 64.7) | 61.2  (56.6, 65.8) | 50.0  (42.5, 57.6) | 59.4  (54.6, 64.1) |
| Diabetes† | 12.7  (7.2, 18.2) | 6.4  (3.8, 9.0) | 14.7  (9.0, 20.3) | 7.9  (0.5, 10.5) | 16.7  (13.7, 19.7) | 10.0  (7.4, 12.5) | 11.4  (9.6, 13.1) | 4.7  (3.4, 5.9) |
| History of myocardial infarction | 4.9 (3.8, 5.9) | 1.6 (1.1, 2.2) | 4.7 (3.5, 6.0) | 2.4 (1.4, 3.4) | 4.1 (2.4, 5.8) | 3.8 (1.8, 5.8) | 3.1 (1.7, 4.5) | 1.3 (0.1, 1.7) |
| History of stroke | 2.9 (1.7, 4.1) | 1.9 (0.7, 3.0) | 3.4 (2.9, 3.9) | 1.2 (0.1, 1.5) | 2.4 (1.8, 3.0) | 2.9 (1.0, 4.9) | 2.6 (1.7, 3.5) | 1.0 (0.6, 1.4) |
| **Cardiovascular risk factors** |  |  |  |  |  |  |  |  |
| Systolic BP (mm Hg), mean (95% CI) | 130.2  (128.9, 131.5) | 124.2  (122.9, 125.7) | 123. 1  (120.5, 125.8) | 119.8  (118.3, 121.4) | 127.3  (125.6, 128.9) | 122.9  (120.2, 125.6) | 129.2  (127.9, 130.5) | 124.0  (122.4, 125.7) |
| Diastolic BP (mm Hg), mean (95% CI) | 83. 2  (82.0, 84.3) | 79.1  (78.2, 80.0) | 79.0  (77.7, 80.2) | 76.2  (75.6, 76.7) | 80.8  (79.8, 81.7) | 77.9  (77.0, 78.9) | 81.5  (80.8, 82.3) | 78.4  (77.8, 78.9) |
| BMI (kg/m^2^), mean (95% CI) | 28.7  (27.6, 29.9) | 26.8  (26.1, 27.6) | 27.7  (26.5, 28.9) | 26.4  (25.6, 27.2) | 25.6  (24.7, 26.5) | 24.4  (23.9, 24.9) | 27.2  (26.7, 27.6) | 26.6  (25.5, 27.6) |
| BMI≥30, % (95%CI) | 37.2  (29.2, 45.1) | 24.8  (20.2, 29.2) | 30.9  (22.8, 39.0) | 20.8  (15.2, 26.3) | 17.2  (12.1, 22.4) | 12.5  (9.7, 15.3) | 25.7  (22.4, 29.0) | 20.5  (16.0, 25.0) |
| Alcohol use, % (95%CI) | 21.6  (8.6, 34.5) | 25.0  (12.0, 38.1) | 16.0  (10.6, 21.4) | 15.1  (8.8, 21.3) | 12.4  (5.0, 19.8) | 5.5 (1.3, 9.7) | 26.3  (13.9, 38.7) | 25.7  (13.6, 37.8) |
| Tobacco use, % (95%CI) | 4.1  (2.0, 6.2) | 3.7  (2.7, 4.8) | 9.7  (5.1, 14.3) | 8.2  (4.8, 11.6) | 9.4  (6.7, 12.0) | 9.2  (6.3, 12.0) | 14.8  (8.0, 21.7) | 10.2  (6.1, 14.4) |
| **BP measured in past 12 months,** % (95%CI ) | 64.3  (51.3, 77.2) | 56.2  (46.4, 65.9) | 77.4  (72.7, 82.1) | 74.1  (70.2, 78.0) | 75.2  (62.0, 88.4) | 45.6  (10.0, 81.1) | 73.3  (61.3, 85.7) | 78.4  (69.3, 87.4) |

*on antihypertensives or BP≥140/90 (using the mean of the second and third BP measurements or imputed BP measurements where missing) at time of screening

†includes type1, type 2, and gestational diabetes

**Table S6: Cardiovascular risk profile and medication use in pregnant vs non-pregnant women in the MMM 2019 population (age standardised to MMM 2019 pregnant population) (sensitivity analysis excluding all women with age>45yrs)**

| **Comorbidity/ cardiovascular risk factor** | **Pregnant** | **Not pregnant** |
| --- | --- | --- |
| **N** | 15 561 | 340 324 |
| **Comorbidities,** % (95% CI) |  |  |
| Hypertension* | 13.8 (11.5, 16.1) | 15.5 (10.7, 20.2) |
| Proportion of hypertensives on medication | 39.5 (34.0, 45.0) | 38.4 (25.3, 51.4) |
| Proportion of those on medication with BP<140/90 mm Hg | 67.2 (61.7, 72.7) | 66.0 (63.2, 68.8) |
| Diabetes† | 3.6 (2.8, 4.9) | 3.4 (1.9, 6.0) |
| History of myocardial infarction | 1.2 (0.9, 1.7) | 2.1 (0.9, 4.8) |
| History of stroke | 1.1 (0.7, 1.7) | 2.0 (0.8, 4.1) |
| **Cardiovascular risk factors** |  |  |
| Systolic BP (mm Hg), mean (95% CI) | 115.3 (113.0, 117.5) | 115.6 (112.7, 118.4) |
| Diastolic BP (mm Hg), mean (95% CI) | 73.7 (71.6, 75.8) | 74.5 (72.5, 76.6) |
| Alcohol use‡, % (95%CI) | 9.2 (4.6, 17.7) | 12.5 (6.8, 21.9) |
| Tobacco use‡, % (95%CI) | 4.5 (3.2, 6.2) | 6.2 (3.9, 9.6) |
| **BP measured in past 12 months,** % (95%CI) | 56.7 (41.6, 70.5) | 42.3 (24.5, 62.4) |

*on antihypertensives or BP≥140/90 (using the mean of the second and third BP measurements or imputed BP measurements where missing) at time of screening

† includes type1, type 2, and gestational diabetes

‡ This was not a pregnancy specific survey; questions were “Do you use tobacco?” and “Do you consume alcohol?”

**Table S7: Cardiovascular risk profile in the pregnant MMM 2019 population in those who have hypertension (see text for details) and in those who have no hypertension (age standardised to MMM 2019 total pregnant population) (sensitivity analysis excluding all women with age>45yrs)**

| **Comorbidity/ cardiovascular risk factor** | **Pregnant women**  **(n=15 561)** | |
| --- | --- | --- |
|  | **With hypertension** | **Without hypertension** |
| **Comorbidities** |  |  |
| Diabetes*, % (95% CI) | 7.6 (5.8, 9.4) | 2.8 (1.9, 3.7) |
| History of myocardial infarction | 2.5 (1.4, 3.7) | 1.0 (0.7, 1.4) |
| History of stroke | 2.5 (1.4, 3.6) | 0.8 (0.4, 1.3) |
| **Cardiovascular risk factors** |  |  |
| Systolic BP (mm Hg), mean (95% CI) | 134.7 (132.9, 136.4) | 112.1 (109.7, 114.6) |
| Diastolic BP (mm Hg), mean (95% CI) | 87.1 (86.1, 88.2) | 71.6 (69.5, 73.7) |
| Alcohol use†**,** % (95% CI) | 14.7 (5.4, 23.9) | 8.4 (2.4, 14.4) |
| Tobacco use†**,** % (95% CI) | 7.6 (4.5, 10.6) | 4.1 (2.8, 5.4) |
| **BP measured in past 12 months,** % (95% CI) | 59.6 (48.5, 70.7) | 56.2 (40.2, 72.2) |

*includes type1, type 2, and gestational diabetes

†This was not a pregnancy specific survey; questions were “Do you use tobacco?” and “Do you consume alcohol?”

**Supplemental figure**

**Figure S1: MMM 2019 Data Capture Form**
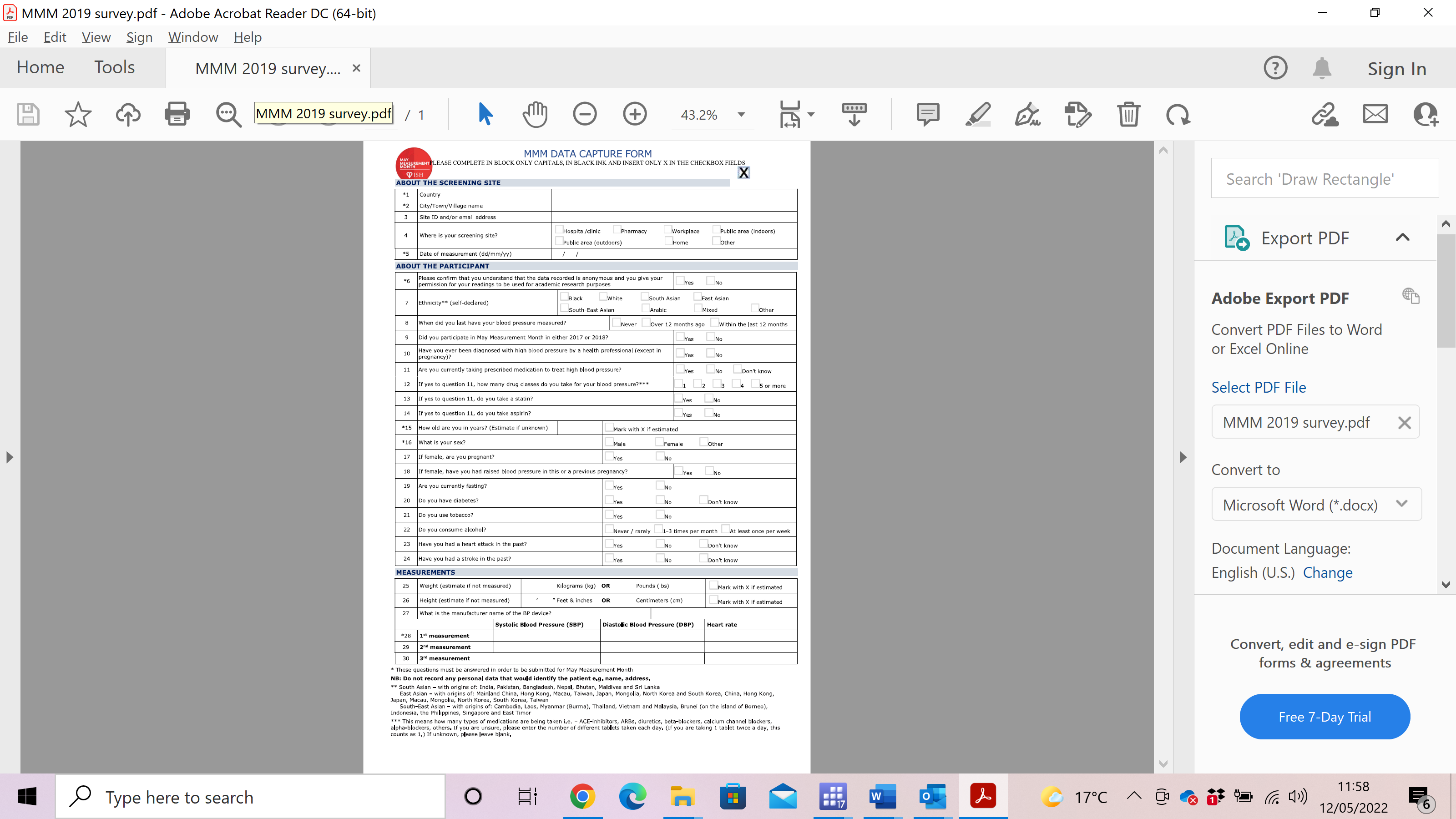

Supplement: Supplementary file 1 [file hyp-81-2298-s001.docx]
